# What *and* Where: Location-Dependent Feature Sensitivity as a Canonical Organizing Principle of the Visual System

**DOI:** 10.3389/fncir.2022.834876

**Published:** 2022-04-12

**Authors:** Madineh Sedigh-Sarvestani, David Fitzpatrick

**Affiliations:** Functional Architecture and Development of Cerebral Cortex, Max Planck Florida Institute for Neuroscience, Jupiter, FL, United States

**Keywords:** retinotopy, cortical maps, topography, visual field specializations, cross-species, tree shrews

## Abstract

Traditionally, functional representations in early visual areas are conceived as retinotopic maps preserving ego-centric spatial location information while ensuring that other stimulus features are uniformly represented for all locations in space. Recent results challenge this framework of relatively independent encoding of location and features in the early visual system, emphasizing location-dependent feature sensitivities that reflect specialization of cortical circuits for different locations in visual space. Here we review the evidence for such location-specific encoding including: (1) systematic variation of functional properties within conventional retinotopic maps in the cortex; (2) novel periodic retinotopic transforms that dramatically illustrate the tight linkage of feature sensitivity, spatial location, and cortical circuitry; and (3) retinotopic biases in cortical areas, and groups of areas, that have been defined by their functional specializations. We propose that location-dependent feature sensitivity is a fundamental organizing principle of the visual system that achieves efficient representation of positional regularities in visual experience, and reflects the evolutionary selection of sensory and motor circuits to optimally represent behaviorally relevant information. Future studies are necessary to discover mechanisms underlying joint encoding of location and functional information, how this relates to behavior, emerges during development, and varies across species.

## Introduction

In many parts of the brain, neurons are sensitive to changes in small parts of the visual field, sampled by the retina in the back of the eye. Often, nearby neurons in the brain signal changes in nearby visual field locations. This mapping of nearby locations on the retina onto nearby regions of the brain is referred to as retinotopic organization. Of course, neural circuits encode many other aspects of visual stimuli aside from spatial location, including features critical for different visuo-motor behaviors such as identifying objects, tracking or otherwise interacting with objects, and moving through the world. How are these representations of visual space organized in relation to other stimulus features? In early visual areas that exhibit highly organized retinotopic maps, understanding this relationship has focused on the fine scale organization of cortical circuits that ensures uniform coverage for stimulus features across visual space (Swindale et al., 2000; White et al., 2001; Bosking et al., 2002; Buzás et al., 2003; Yu et al., 2005), rather than how response properties might differ for different regions of visual space. Likewise, in higher visual areas that are specialized to represent specific stimulus properties, the spatial locations associated with the stimulus attributes of a given area are only recently being characterized (Groen et al., 2021).

Recent findings open the door to a new perspective on the encoding of visual space and other features by cortical circuits, one that emphasizes specialization of cortical circuits for processing different stimulus features in different regions of visual space. Here we review the evidence for this location-dependent coding across various stages and scales of the visual system. This includes location-biased sampling of functional features in the output ganglion cell layer of the retina, systematic variation of functional properties within conventional retinotopic maps, and novel periodic retinotopic transforms that dramatically illustrate the tight linkage of feature sensitivity, spatial location, and specialized cortical circuitry. The location-dependent feature sensitivity exhibited at the cortical level appears consistent with the positional regularities in naturally experienced visual input, produced by the statistics of the environment as well as the statistics of bodily movements. Furthermore, a comparative analysis of the published literature supports a species-specific correlation between retinotopic location and functional feature processing across the parallel streams, the fundamental organizing scheme of the visual system originally defined based solely on distinct functional feature sensitivity across areas.

Based on our review and analysis of this evidence, we propose that the joint encoding of ego-centric spatial location and functional features is a canonical organizing principle of the visual system, likely reflecting evolutionary pressures that shape neural circuitry to optimally represent regularities in functionally relevant visual information. One implication of this framework is an increased need to study structural and functional properties of the developing and mature retina in order to understand the location-dependent encoding produced by specialized retinal sampling. Another implication is a need to revise experiments that investigate the representation of spatial location and functional feature sensitivities separately, since neural circuitry imposes inextricable dependencies between these features. We present other implications of this new framework and conclude with a discussion of how retinotopic specializations can be used to establish an ethological understanding of neural encoding, within experimental and computational studies of the visual system.

## Visual Inputs Received by the Retina Exhibit Location-Dependent Statistics

**FIGURE 1:**
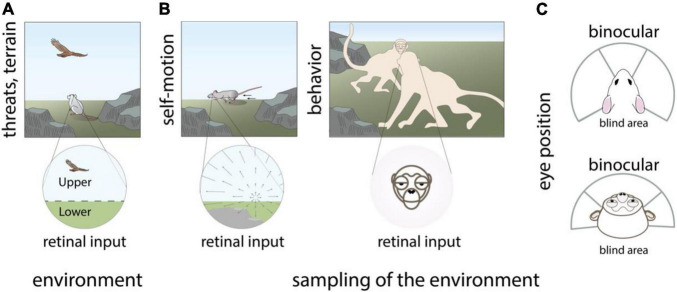
Visual inputs received by the retina exhibit location-dependent statistics. Topographic variations in visual inputs to the retina are species-specific and arise from **(A)** non-uniform statistics of the visual environment such as predators that appear overhead, **(B)** Non-uniform behavioral sampling of the visual environment as produced by self-motion of the eyes, head, and body, as well as other behaviors including social communication, **(C)** the position of the eyes in the head and resulting extent of the binocular visual field. Panels **(A,B)** modified with permission from © 2021 Dina Popovkina.

The first place to look for evidence of joint encoding of retino-centric location and functional features is obviously the visual inputs to the retina, the first stage of visual processing and the interface between the environment and the visual system. Even without specific measurements, one can safely assume that location and stimulus features are not randomly arrayed in the visual environment, leading to statistical biases in the distribution of visual stimuli that fall on different parts of the retina (Simoncelli and Olshausen, 2001; Geisler, 2008). For instance, predators of mice regularly appear overhead in the blue sky. This produces non-uniform features with distinct statistics and behavioral salience, such that the upper and lower visual field of small rodents exhibit distinct color distributions (Qiu et al., 2021; Figure 1A).

Other sources of non-uniform retinal input arise from the animal’s sampling of the environment through bodily structure, bodily movements, and other natural behaviors including social communication. Optic-flow related to self-motion of the eyes and body produces distinct structured patterns that can differ across regions of the retina (Angelaki and Hess, 2005; Calow and Lappe, 2007; Bigge et al., 2021). Similarly, movements of the head, especially when not compensated by stabilizing eye movements (Meyer et al., 2020; Michaiel et al., 2020) can produce different spatiotemporal visual inputs in different locations of each retina, and across the left and right retinae. Social behaviors, such as facing other animals during vocal communication in primates produce a high probability for faces in the central as compared to the peripheral visual field (Figure 1B). The position of the eyes, which are in front of the head in primates and predator animals including cats, or on the side of the head in rodents, tree shrews, and many prey animals including rabbits, influences the relative size of the visual field that is binocular vs. monocular (Figure 1C). In addition, convergence and divergence of the eyes can impact the shape and content of the visual field during natural behaviors (Wallace et al., 2013; Holmgren et al., 2021; Johnson et al., 2021). Altogether, these factors influence the relative structure of visual input across the temporal and nasal regions of the left and right retinae. Many prey animals also tend to have eyes close to the ground, creating differences in visual inputs between their lower and upper visual field that is more exaggerated compared to upright primates, arboreal, or flying animals.

## Location-Dependent Feature Sensitivity in the Retina and Early Visual Cortex

Given the positional regularities present in their inputs, it stands to reason that retinal circuits exhibit corresponding specializations and convey these to central visual targets. Here we discuss established and recent evidence in support of topographic specializations in the retina and early visual areas of rodents and primates, the two species with the most published data on this topic.

**FIGURE 2:**
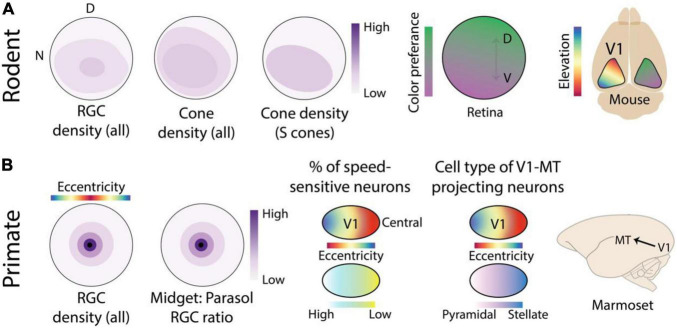
Location-dependent feature sensitivity in the retina and early visual cortex of rodents and primates. **(A)** Averaged across all RGC cell types, the rodent retina has a slightly higher density in the ventral retina that receives input from the upper visual field, however this specialization is relatively much shallower than the foveal specialization of primates shown in panel **(B)**. In addition, the distribution of cone photoreceptors shows slight topographic anisotropies. However, when considering the distribution of short-wavelength (s) opsins alone, a much deeper gradient can be seen across the dorsal-ventral elevation axis (Nadal-Nicolás et al., 2020). This produces a location-dependent encoding of spectral sensitivity in the output pathways of the retina that is inherited by neurons in mouse V1 (Rhim et al., 2017). **(B)** Primate retinas exhibit foveal specialization, characterized by a sharp drop-off in RGC density with increasing distance from the center of the retina or equivalently, with increasing eccentricity. The ratio of midget and parasol RGC cell types also differs greatly with eccentricity, producing distinct functional response properties across the retina (Dacey, 1993). In marmoset V1, several subtle eccentricity-based specializations have been reported including a peripheral specialization for motion processing (Yu and Rosa, 2014; Yu et al., 2015). More notably, MT projecting neurons in layer 3C exhibit distinct cell types as a function of eccentricity (Mundinano et al., 2019). Retina figures are schematics designed to show topographic gradients, therefore the color bar is normalized and only indicates relative “high” and “low” ends of the scale.

The mouse retina was long assumed to exhibit topologically uniform functional feature sensitivities, but advanced genetic approaches have revealed distinct retinal specializations in this animal (Figure 2A; Bleckert et al., 2014; El-Danaf and Huberman, 2019; Heukamp et al., 2020). The best studied variation concerns the non-uniform distribution of S and M sensitive cone photoreceptors (Szél et al., 1992; Röhlich et al., 1994; Baden et al., 2013; Nadal-Nicolás et al., 2020). This pattern reflects optimal sampling of environmental statistics from the mouse’s perspective (Baden et al., 2013; Qiu et al., 2021). In addition to environmental statistics, traces of self-motion are also apparent across the mouse retina. Motion direction selectivity in a subset of RGC neurons is tightly related to retinal location in a manner that mirrors the visual inputs during forward self-motion (Sabbah et al., 2017). Presumably, this topographic variation aids the animal in estimating its own motion from visual inputs, but this hypothesis has yet to be tested. Topographic specializations have also been discovered in several retinal ganglion cell (RGC) subtypes with unique functional properties (Bleckert et al., 2014; Warwick et al., 2018; Heukamp et al., 2020), although their contribution to behavior (Johnson et al., 2021) is not yet understood.

Early visual areas preserve the global topography of the retina. This implies that topographic variation in feature sensitivity in the output of the retina may also be preserved in early visual areas. This is indeed the case for location-dependent color encoding in the mouse retina, which has been shown (Rhim et al., 2017) to be preserved in mouse V1 (Figure 2A). The cortical representation of the lower visual field in V1 is more sensitive to middle (green) and the representation for the upper visual field is more sensitive to short or UV wavelengths, corresponding not only to upstream retinal specializations but also to behavioral chromatic sensitivity across the visual field (Denman et al., 2018).

Given the joint encoding of location and motion direction in the signal relayed by the retina (Sabbah et al., 2017), one might expect to find location-dependent encoding of motion direction or other features related to self-motion in early visual areas. Several groups (de Malmazet et al., 2018; Li et al., 2020) have recently reported the joint encoding of spatial location and motion direction in the mouse superior colliculus (SC), with structures that could support the encoding of self-motion induced optic flow. However, these reports produced conflicting patterns of the retinotopic specialization and corresponding encoding for optic flow. An exhaustive effort to clarify these findings (Chen et al., 2021) by combining multiple measurement modalities, visual stimuli, behavioral states, and sampling large regions of the retinotopic map found no evidence of correlated spatial location and motion direction encoding in mouse SC. Characterizing species-specific patterns of self-motion (Carriot et al., 2017; Bigge et al., 2021) or experienced optic flow would help to resolve whether positional regularities in direction of motion produced by optic-flow are represented in the visual system beyond the retina (Sabbah et al., 2017). Recording neural activity across multiple regions of freely behaving animals will further help to determine specializations related to visual processing in the context of bodily movements and navigation (Saleem, 2020).

We note that not all location-dependent feature specializations must be inherited from the retina, as precise synaptic wiring in the retina to cortex or retino-tectal pathways can produce these specializations *de novo*. For instance, by definition, binocular sensitivity in the central visual field (Berman et al., 1982) cannot be inherited from the retina and must be computed by combining information from the two retinae. The source of other location-dependent feature specializations in V1, such as an increased sensitivity to coherent motion in the lower visual field (Sit and Goard, 2020), are harder to parse from basic principles and require careful synaptic examination of topographic variation in cortical circuitry.

Long before the introduction of the mouse as a model organism of the visual system, retinal specializations were noted in larger animals including cats, various non-human primates, and humans. In fact, the most obvious retinal specialization is the fovea of humans, some non-human primates (Bringmann et al., 2018), and some non-mammalian species. The fovea (Figure 2B) is a small retinal area densely packed (Wässle et al., 1989; Curcio and Allen, 1990) with photosensitive cones and retinal ganglion cells (RGCs) with small receptive fields, that provide specialized sensitivity to color and fine features in the most central location of the visual field (Wassle and Boycott, 1991; Sinha et al., 2017).

In addition to cell density and corresponding receptive field size, RGC cell type also varies across the central-peripheral gradient of the primate retina. Midget cells have small and color sensitive receptive fields with slow temporal filtering whereas parasol cells have large, color-blind, receptive fields with fast temporal filtering (Shapley and Hugh Perry, 1986; Kolb and Marshak, 2003). Although the absolute numbers of both cell types decrease with distance from the fovea, their ratio changes as a function of eccentricity (Dacey and Petersen, 1992; Dacey, 1993). Near the fovea, midget cells outnumber parasol cells by several to 1, estimated to be as high as 30 to 1 in humans (Dacey and Petersen, 1992), whereas in the periphery the numbers are nearly balanced. This eccentricity-dependent contribution of midget and parasol cells is preserved along the retino-thalamic-cortical pathway to V1 (Azzopardi et al., 1999). Altogether, such topographic variations in cone and RGC density and cell type create feature-encodings specialized for color and fine details in the central visual field and coarse moving patterns in the peripheral visual field of primates (Heukamp et al., 2020). It is possible that other retinal specializations exist in the primate retina, related to other environmental or self-motion related regularities in visual input. However, the relative lack of modern genetic tools for cell-type specific interrogation of primate retinal circuits has produced a dearth of knowledge on topographic variation in primate (Peng et al., 2019; Yan et al., 2020) vs. rodent retinas.

It is relatively difficult to study topographic variation in primate, vs. rodent, cortex for two reasons. First, the brains of most primates exhibit folds which make optical imaging of the full retinotopic map of a visual area rather difficult. Secondly, the genetic and viral techniques that allow optical imaging in rodents do not necessarily translate to primates, although primate-specific viral vectors have recently proven successful (Seidemann et al., 2016; Li et al., 2017). Nonetheless, topographic organization of feature encoding has been studied using electrophysiology in the folded brains of larger primates, revealing feature encodings that vary primarily across the central-peripheral eccentricity axis (Adams and Horton, 2003; Yu et al., 2015).

In primate V1, as one moves from central to peripheral locations of the retinotopic map, there are major changes in properties related to spatial acuity and binocularity (Daniel and Whitteridge, 1961; Adams and Horton, 2003), and more subtle changes related to motion processing (Orban et al., 1986; Yu et al., 2010, 2015; Yu and Rosa, 2014). This gradient reflects the topographical distribution of the different RGC types in primate retina (Azzopardi et al., 1999; Figure 2B) as well as the position of the eyes. But as discussed above, specializations in cortical areas are not always simply inherited from incoming input and may become exaggerated or develop *de novo* based on non-uniform anatomical, structural, or molecular properties of neurons in different portions of the retinotopic map. This has been nicely demonstrated in marmoset area MT, which exhibits distinct connections with other visual and non-visual areas in different parts of its retinotopic map (Figure 2B). Regions in the central representation are connected to other visual areas like V1, V2, V3, and V4 whereas regions in the far peripheral representation receive inputs mainly from peripheral V1 and area prostriata in the retrosplenial cortex (Palmer and Rosa, 2006). Zooming in further, remarkable retinotopic specializations exist within the V1-MT projection (Mundinano et al., 2019). MT-projecting neurons, in layer 3C of V1, in the central regions of the retinotopic map are mostly spiny stellate whereas neurons in the peripheral representation are mostly pyramidal. Distinct connectivity between the central and peripheral representation of visual areas and the rest of the brain have also been observed in other cortical areas of the marmoset (Majka et al., 2019) as well as in the visual cortex of humans (Griffis et al., 2017; Sims et al., 2021). Future studies using optical imaging in smooth-brained primates may reveal additional topographic specializations in early cortical areas.

## Complex Retinotopic Transforms Highlight Location-Dependent Feature Sensitivity

We have shown that within individual visual areas, functional feature sensitivity exhibits location-dependence in a manner that may reflect efficient representation of positional regularities in visual input (Simoncelli and Olshausen, 2001; Geisler, 2008). In this view, there is no *a priori* reason why all visual areas should exhibit complete topographic representations of retinal location that preserves the planar layout of the retina. Instead, the coverage and geometry of retinotopic maps is inherently related to the functional features encoded within a visual area. Here we review recent discoveries of retinotopic maps that deviate from conventional topographic representation of retinal location and highlight the ubiquitous location-dependent feature sensitivity in the visual system. We use this data to suggest that functional feature encodings may influence, or co-emerge with, retinotopic representations. Other factors, including the developmental time-course of cortical areas have also been proposed to influence the coverage and geometry of retinotopic maps (Rosa, 2002).

Throughout this and the following sections, we use published definition of visual areas, which are usually demarcated by a combination of anatomy, histochemistry, and functional measurements including visual field coverage. However, we remind readers that the rules of area delineation differ among species as inherent differences in their visual systems prevents application of identical rules. For instance, higher order visual areas in the mouse cover a small region of the visual field whereas extrastriate areas in the primate can cover the entire visual field, although in some cases there are significant biases in coverage. We also caution that reports of limited visual field coverage in any species may be due to methodological limitations or subsampling. However, as we explain in the following section, location-dependent feature sensitivity can be seen across the entire visual system of primates and rodents. Therefore, the precise definition of visual area boundaries does not undermine our central proposal that these features are not independently processed by visual circuits.

**FIGURE 3:**
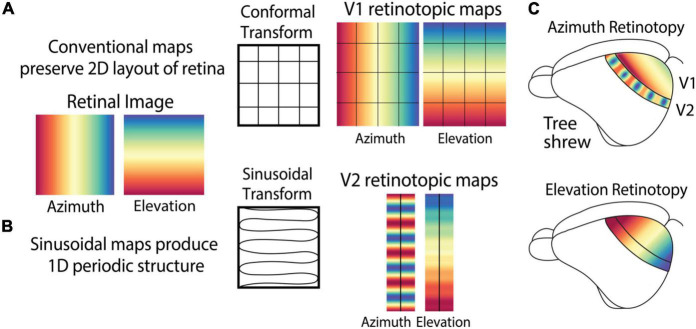
Retinotopic maps in the cortex can exhibit complex patterns that do not maintain the topographic layout of the retina. **(A)** Maps of retinotopic spatial location in V1 of most animals, including tree shrews, exhibits a conformal transform that preserves 2D layout of the retina including the orthogonal relationship between azimuth and elevation. **(B)** V2 of tree shrews exhibits a sinusoidal retinotopic transform that combines azimuth and elevation to produce a periodic structure. **(C)** Azimuth and elevation retinotopic maps in tree shrew V1 and V2.

The conventional description of retinotopic maps is “conformal”: a point-to-point relationship with the retina that preserves both local relationships as well as the global layout of the retinal image (Schwartz, 1977; Sereno et al., 1994; Adams and Horton, 2003; Garrett et al., 2014), albeit with certain deformations (Wolf et al., 1994; Ta et al., 2014), and magnifications (Figure 3A). In V1 of all studied animals, retinotopic maps are simple in that they preserve the orthogonal axes of the retina. As in tree shrew V1, the azimuth axis of the retina is usually mapped along the medial/lateral axis of the brain, and the orthogonal elevation axis is mapped along the orthogonal anterior/posterior cortical axis (Bosking et al., 2000; Manger et al., 2002; Garrett et al., 2014). Deviations from the conformal mapping in V1 were noted in higher visual areas of primates and cats but were often discussed in terms of a “discontinuity” within an otherwise continuous map of the visual field (Sanides and Albus, 1980; Wolf et al., 1994; Roe and Ts’o, 1995). Our recent study (Sedigh-Sarvestani et al., 2021) of the tree shrew visual system showed that retinotopic maps can exhibit all-together different transformations of the visual field that do not preserve the two-dimensional spatial layout of the retina. A recent study (Yu et al., 2020) in the “third tier” visual cortex of primates also reported a non-conformal map, although the particular retinotopic transform was different from our observations in tree shrew V2.

Unlike the conventional retinotopy in V1 of many species, the retinotopic transform in tree shrew V2 can be described as a sinusoidal transform (Figure 3B) that converts the 2D planar layout of the retina to a roughly 1D periodic structure. This transform combines elevation and azimuth by mapping them onto the same elongated axis of V2, no longer preserving their global orthogonal relationship. This produces a simple map of elevation and a periodic map of azimuth, with both maps along the length of V2 (Figure 3C).

The periodic map of retinal location oscillates between representations of the most central regions of the visual field with representation of paracentral regions separated by 10–15 visual degrees. This representation is tightly linked to enhanced sensitivity for binocularity and retinal disparity: small changes in the retinal image viewed by the two eyes that can be used to discriminate distance and depth. This suggests that enhanced binocularity and disparity sensitivity are specialized for the most central regions of the visual field. Furthermore, the representation of this region of the visual field has enhanced interhemispheric connectivity. The correlated periodic pattern in visual field location and feature sensitivity is also reflected in the pattern of callosal terminals, cross-hemispheric projections which carry information about the most central regions of the visual field. Thus, the most central region of visual space specialized with enhanced binocular sensitivity also features enhanced interhemispheric connectivity, which may ensure coherence of bilateral responses in this region of visual space.

**FIGURE 4:**
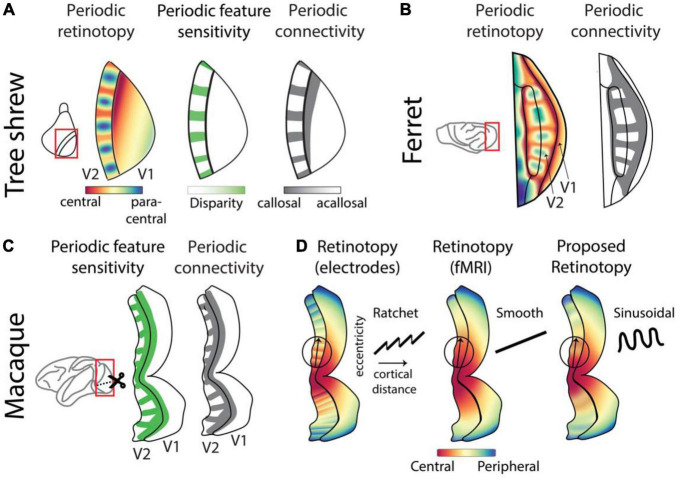
Complex retinotopy highlights location-dependent feature sensitivity across species. **(A)** The complex retinotopy map in tree shrew V2 highlights the joint distribution of spatial location, disparity sensitivity, and anatomical connectivity. Regions of V2 that are sensitive to the most central regions of the binocular visual field are also sensitive to retinal disparity cues and receive callosal inputs which carry information from V1 of the opposite hemisphere (Sedigh-Sarvestani et al., 2021). **(B)** A similar periodic retinotopy, with corresponding periodic pattern of callosal inputs, has been observed in ferret V2 (Manger et al., 2002; Rosa and Manger, 2005). **(C)** Periodic patterns of feature sensitivity, including retinal disparity, are well-known in macaque V2 (Hubel and Livingstone, 1987; Lu et al., 2010; Hubel et al., 2015). Callosal projections exhibit a corresponding periodicity (Olavarria and Abel, 1996), suggesting that retinotopic sensitivity may also exhibit a periodic pattern similar to that in tree shrews or ferrets. **(D)** Reports of retinotopy in macaque V2 differ based on methodology. When measured using invasive single-cell electrodes, retinotopy has been reported as a “ratchet” model (Roe and Ts’o, 1995; Shipp and Zeki, 2002) with smooth increases interleaved with sudden resets in receptive field position. When measured with the coarser resolution of fMRI, smooth maps were reported (Nasr et al., 2016; Li et al., 2019). We propose that optical imaging measurements which combine single-cell resolution with widefield coverage would exhibit sinusoidal retinotopy overlying a smooth gradient, similar to our observations in tree shrews and ferrets.

Therefore, the structured visual input produced by the position of the eyes in the head, as well as the interhemispheric connectivity, are consistent with the enhanced representation of binocular features in the most central regions of the visual field. However, as discussed in the previous sections (Figure 1), such location-dependent encoding can arise due to several other underlying factors. A remarkably similar pattern has been observed (Manger et al., 2002) in secondary areas of the ferret visual cortex (Figures 4A,B) where the sensitivity to retinotopic location and connectivity with the opposite hemisphere vary in a periodic fashion. Future experiments will determine whether functional feature sensitivity varies in a periodic manner tightly connected to retinotopy in ferret secondary visual areas, as it does in the tree shrew (Manger et al., 2002; Rosa and Manger, 2005; Sedigh-Sarvestani et al., 2021).

A similar periodic functional feature map exists in primate V2 (Figure 4C), where the sensitivity to binocularity, disparity, and several other features including color change in a periodic manner along the length of this region (Hubel and Livingstone, 1987; Lu et al., 2010; Hubel et al., 2015). It is not clear whether this functional encoding is correlated with an underlying retinotopic periodicity. However, since callosal projections consistently terminate near the representation of the vertical meridian in multiple species, the presence of periodic bands of callosal terminals in V2/V3 of macaques supports a common periodic retinotopic transform across macaques, tree shrews and ferrets (Rosa and Manger, 2005).

Retinotopy in macaque V2 has been measured using various techniques. Widefield fMRI measurements from V2 that exhibit feature stripes do not exhibit corresponding retinotopic stripes but report a smooth map (Nasr et al., 2016; Li et al., 2019; Figure 4D, middle). However, measurement using single-cell electrode recordings report a non-smooth “ratchet” retinotopy (Roe and Ts’o, 1995; Shipp and Zeki, 2002) at the local scale (Figure 4D, left), where smooth linear projections in spatial location representations are interrupted by sudden “switch backs” at the border of functional feature stripes. We propose that the actual retinotopic map in primate V2 exhibits a sinusoidal structure overlying a smooth map (Figure 4D, right). This sinusoidal retinotopy might appear as a “ratchet” pattern when sampled with single electrodes which lack widefield coverage, and it might appear as a smooth map when measured with fMRI which lacks single-cell resolution. Our proposal, consistent with a previous modeling prediction (Swindale, 2007), can be assessed using optical imaging methods such as calcium imaging that combine high spatial resolution with widefield coverage.

In both tree shrews and ferrets, locations along the two axes of the retina are mapped differently. While the central-peripheral or azimuth axis is mapped in a periodic fashion, the elevation axis is mapped in the conventional smooth manner. Future work may reveal a functionally significant rationale for this mapping. Alternatively, it may be a trivial consequence of a periodic transform optimized for enhanced representation of the central visual field. More broadly, flexibility in the geometry of the retinotopic map may enable the representation of visual information in the most functionally relevant regions of the visual field. Conversely, if parts of the visual field, or one of its two axes, are not utilized in the functional role of a brain area, the retinotopic map should reflect a correspondingly limited representation (Kay et al., 2015).

## Parallel Stream Organization Reveals Species-Specific Joint Encoding of Location and Features Across the Entire Visual System

**FIGURE 5:**
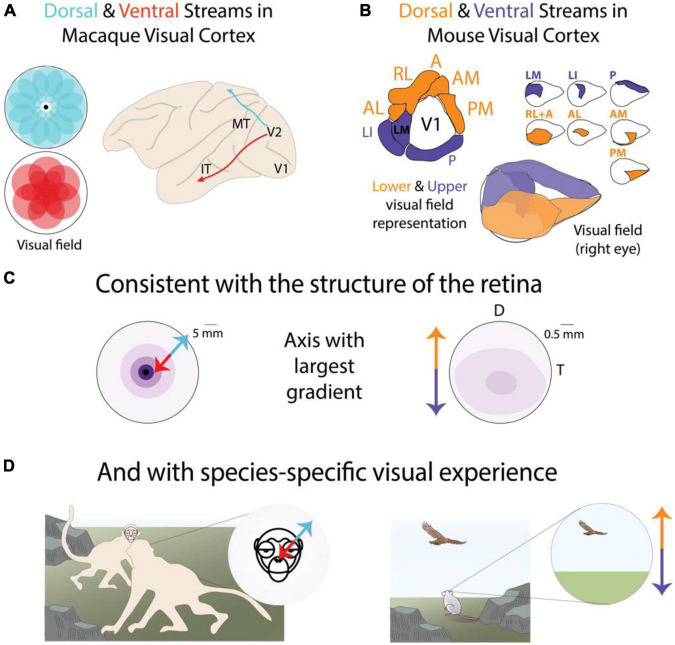
Parallel stream organization reveals species-specific joint encoding of location and features across the entire visual system. **(A)** The macaque functional parallel streams exhibit distinct biases along the central-peripheral axis of the visual field (Yu et al., 2015; Shetht and Young, 2016). **(B)** The mouse functional parallel streams exhibit distinct biases along the elevation axis of the visual field (Garrett et al., 2014; Zhuang et al., 2017). **(C)** Topographical distribution of RGCs in the macaque and mouse retina exhibit their largest variance along different axes (Heukamp et al., 2020). Darker colors indicate higher density. **(D)** The visual behaviors of macaques and mice are broadly consistent with distinct functional relevance of central-peripheral or upper-lower visual fields. Panel modified with permission from © 2021 Dina Popovkina.

We have discussed evidence for joint representation of retinotopic location and functional features in individual visual areas (Figures 1–4). Here we provide evidence for joint encoding of location and features in higher visual areas along the parallel streams that form the rest of the cortical visual system. The dorsal and ventral streams (Figure 5) are considered the fundamental organizing scheme of the primate (Schneider, 1969; Mishkin and Ungerleider, 1982), and more recently the rodent (Wang et al., 2011; Glickfeld and Olsen, 2017; Bennett et al., 2019), visual system. Each stream is composed of multiple visual areas and is distinguished based on several factors including sensitivity to functional features, anatomical connectivity with visual and non-visual areas, and contribution to visuo-motor behaviors.

We further show that, when considered together, dorsal and ventral stream areas exhibit biases in their visual field coverage (Yu et al., 2015; Shetht and Young, 2016) broadly consistent with the functional roles attributed to each stream. Furthermore, we highlight that the location bias is species-specific, with primates exhibiting functional biases along the central-peripheral axis of the visual field and rodents exhibiting biases more pronounced along the lower-upper axis (Figure 5). We begin with a discussion of the primate literature where higher order visual areas and parallel streams were first identified.

The study of parallel streams originated with a focus on functional, not retinotopic, sensitivities. Until recently it was generally assumed that higher visual areas in primates did not exhibit precise retinotopy. Therefore, there is a lack of data on topographic variability in feature sensitivity in higher visual areas. However, recent advances in fMRI technology have revealed ubiquitous location-dependent functional sensitivity in higher order areas of the primate visual system (Hasson et al., 2002; Groen et al., 2021). Multiple retinotopic maps have been reported to correlate with previously known functional specializations in ventral temporal/inferior temporal (IT) cortex for faces, scenes, and buildings. Face-preferring regions tend to overlap the central representation whereas scene and building-preferring regions overlap with the peripheral representation of the visual field (Levy et al., 2001). A similar organization was found in the macaque (Arcaro and Livingstone, 2017a). Some regions also exhibited biases for the upper visual field (Silson et al., 2018), perhaps reflecting a lack of functionally distinct visual information relevant for the detection of faces in the lower visual field. Single neuron recordings in macaques confirmed this central specialization, showing that face-selective neurons had large receptive fields that on average preferred stimuli near the fovea (Gomez et al., 2018). Neurons in area V4, also part of the ventral stream and implicated in color processing, emphasize the “central” 30–40° but have not been found to exhibit representations of the far periphery (Gattass et al., 1988).

In contrast to the central visual field bias noted in several areas of the primate ventral stream, there is some evidence to support a relatively reduced magnification of the fovea and a greater coverage of the peripheral visual field in dorsal stream areas such as MT (Rosa and Elston, 1998), and DM (also known as V6), which exhibits an emphasis of the peripheral visual field (Colby et al., 1988; Galletti et al., 1999; Pitzalis et al., 2006). This is consistent with the distribution of the main ganglion cell types that provide a large fraction of inputs to each stream. Near the fovea, a large proportion of all ganglion cells are midget type whereas in the periphery midget and parasol cells are about equally distributed (Dacey, 1993). This results in the midget-recipient ventral stream receiving inputs biased toward the fovea and the parasol-recipient dorsal stream receiving inputs more evenly distributed across the fovea and periphery.

Thus, the functionally distinct parallel streams of the primate visual system appear to exhibit distinct retinotopic biases consistent with their inputs and functional sensitivities. However, there are notable exceptions to this general trend. One exception, as mentioned above, is the peripheral visual field preference of modules within IT, that respond robustly to scenes or buildings (Hasson et al., 2002; Lafer-Sousa and Conway, 2013). Another exception is the relatively larger representation of the central visual field in area LIP, which belongs to the dorsal stream (Ben Hamed et al., 2001).

The parallel streams in the mouse visual cortex have been largely defined by anatomical features (Wang et al., 2011; Glickfeld and Olsen, 2017), and original studies reported relatively subtle differences in functional feature sensitivity (Andermann et al., 2011; Marshel et al., 2011; Juavinett, 2014), when compared to functional specializations in higher visual areas of primates. More recent work (Sit and Goard, 2020; Yu et al., 2021) has shown some differences in feature encoding, as well as in developmental time courses (Murakami et al., 2017; Smith et al., 2017; Salinas et al., 2020), between areas in the dorsal and ventral streams. Area LM, designated to the ventral stream, exhibits higher fidelity representations of texture compared to area AL in the dorsal stream. AL on the other hand is relatively more sensitive to motion (Yu et al., 2021). As proposed by recent modeling studies (Bakhtiari et al., 2021), such feature differences are broadly consistent with the ventral stream participating in recognition and the dorsal stream participating in movement related behaviors, similar to functions attributed to the primate streams.

Despite the sparsity of current evidence for robust feature specializations, the mouse dorsal and ventral streams exhibit clear and pronounced retinotopic biases (Figure 5B). Areas in the dorsal stream collectively tile the lower visual field whereas areas in the ventral stream tile the upper visual field (Zhuang et al., 2017). This retinotopic bias is consistent with the topographic variations (Heukamp et al., 2020) in the mouse retina (Figure 2A), which largely occur along the elevation axis. It is also consistent with location-dependent encodings observed in individual higher visual areas. Specifically, the representation of the lower visual field across the mouse visual system exhibits greater sensitivity to coherent motion (Sit and Goard, 2020), as well as greater sensitivity for binocular disparity (La Chioma et al., 2019) both within and across individual areas. Both location-dependent representations likely reflect natural image statistics related to visual processing in peri-personal or near visual space, which coincides with the lower field in low-lying animals like mice. Therefore, retinotopic separation of functionally distinct visual circuits appears to be the major organizational scheme of the visual system of both primates and rodents.

But why does the main axis of this joint location-feature encoding differ between species (Figures 5A,B)? First, it reflects the main axis of change in the topographical distribution of RGCs in the retina (Figure 5C). Whereas the foveal specialization in macaques exhibits a gradient largely along the central-peripheral axis, the RGC distribution in the mouse retina has a larger deviation along the elevation axis. More importantly, these axes in the retina and cortex can be explained in terms of the visual environment and behaviors of each animal (Figure 5D). The egocentric visual world of a ground-dwelling prey animal contains distinct functionally relevant information, environmental statistics (Qiu et al., 2021), and distance to objects (La Chioma et al., 2019), compared to the upper visual field. On the other hand, the egocentric visual world of the upstanding and social primate contains detailed cues relevant for social communication and order. Therefore, the organization of the visual system of each species reflects the efficient representation of the geometry and positional regularities of their behaviorally relevant visual experience.

It is important to point out that behaviorally relevant visual experience is not always the most frequent or likely visual experience. Therefore, location-dependent feature selectivity patterns may not reflect the statistical structure of the most likely inputs in order to efficiently represent the most relevant inputs, as seen in grasshopper auditory neurons (Machens et al., 2005). Outside of obvious threat or salient environmental events, it is difficult to know which sensory inputs carry more behavior relevance, but a minimal criterion may be the utility to predict future sensory inputs (Palmer et al., 2015; Chalk et al., 2018). While in many situations the best prediction about the future can be estimated from the past, this is not always the case. Therefore, efficient representation of behaviorally relevant inputs may come at the cost of inefficient representation of more frequent but less relevant inputs. This suggests that the use of behaviorally relevant stimulus paradigms is critical for testing theories regarding efficiency of the neural code.

In addition to efficient encoding of salient visual inputs, another explanation for location-dependent feature sensitivity, and other multiplexed inter-dependent feature encodings, may be efficient decoding of sensory inputs for behavioral control. In other words, encoding the multi-dimensional statistics of salient visual inputs may not only confer energetic advantages, it may also have computational benefits such as increasing the accuracy of information available to downstream sensory-motor circuits (Macellaio et al., 2020).

## Implications for Future Studies of the Visual System

The evidence highlighted so far demonstrates a new framework for the organization of the visual system where information about spatial location and functional features are jointly encoded throughout the visual system, in a manner that reflects the specific visual experience of different species. In this view, the geometric distribution of functionally relevant information in the egocentric visual field shapes sensory encoding in the retina and throughout the visual system. A major implication is that our understanding of the structure and function of the visual system is constrained by our understanding of the structural and functional properties of the retina in the context of species-specific visual experience. In the past two decades, several groups have contributed to a growing understanding of the properties of the mouse retina, how these properties reflect the visual experience of mice, and how functionally relevant retinal sampling is communicated to the rest of the visual system (Dhande and Huberman, 2014; Baden et al., 2016; Heukamp et al., 2020). Similar knowledge in other model organisms used in systems-level visual neuroscience pales in comparison. This is partially due to the suite of genetic tools available in mice, but it also reflects a prevailing assumption that functionally simple visual inputs gain complexity only through discrete transitions across each area of the visual cortical hierarchy. Our framework suggests, in addition, that visual information is already functionally specific at the retinal stage and gains further complexity not only across hierarchical areas but also within each area. Consequently, we believe there is a need for greater emphasis on characterization of the visual experience and retinal properties of tree shrews, ferrets, cats, primates, and other model organisms used in vision research.

Specifically, additional research is needed to determine the role of internal and externally driven visual experience in development of retinal specializations. In this review we suggest that retinal specializations, such as topographic biases in the density of specific functional classes of RGC cells, are specific to and consistent with the behaviorally relevant visual experience of animals. However, in most cases we do not know if post-natal visual experience plays any causal role in the formation of retinal specializations in mice or in any other species. A further complication is spontaneously generated “visual experience.” Even before the onset of visual experience during development, activity in different locations of the retina exhibits distinct properties due to the presence of spatiotemporally structured spontaneous activity referred to as “retinal waves” (Feller, 1999; Wong, 1999). It has been suggested (Pratt et al., 2016; Ge et al., 2021) that retinal waves act as a simulacrum of future structured visual experience and serve to scaffold optimal visual circuitry. Cross-species measurement and manipulation of retinal waves will produce further insight into the causal role of this source of early activity in the species-specific organization of the visual system. Further work is also needed to understand the role of post-natal visual experience on the formation of location-dependent specializations in cortical areas (Arcaro and Livingstone, 2021, 2017b).

A second implication is that retinotopic maps can and should be utilized to elucidate the functional properties of visual areas. Most circuit-level studies of the visual system utilize retinotopic measurements primarily for practical matters such as delineating area borders or determining regions of interest for neural recording. However, the retinotopic map itself is rarely considered in attempts to decipher neural computation. Furthermore, many studies report neural encoding in a limited part of the retinotopic representation of a visual area. This practice is problematic in light of retinotopic specializations that exhibit distinct functional encoding, structural connectivity, anatomical, and molecular properties across the retinotopic map of the same visual area. A poor understanding of the retinotopic map, or reliance on measurements from only a single region of the map, could mask potential specializations and produce conflicting reports attributed to a particular cortical region. Furthermore, even at scales far smaller than an entire visual area, neural encoding for location and features needs to be studied together since they are inherently processed together in the brain. For instance, the visual signal received by subcortical and cortical areas will contain correlated information about spatial location and functional features due to non-uniform retinal sampling, even when the externally controlled visual input has uniform features at all locations of the display monitor.

The last implication regards computational models of the visual system, which are increasingly used to test theories about brain and behavior that are currently intractable with experimental approaches (Ponce et al., 2019; Richards et al., 2019; Bakhtiari et al., 2021; Lindsay, 2021; Mineault et al., 2021). The large majority of neural networks used to model the visual system (Schrimpf et al., 2018), including convolutional neural networks specifically modeled after the ventral stream and used to recognize object categories (Lindsay, 2021), lack topographic variation in feature encoding. As we have discussed, location-dependent feature sensitivity appears to be a core organizing principle of the entire visual system, influencing anatomy and connectivity, signal transformations, and ultimately behavior. Therefore, the lack of location-dependent filters in computational models hampers their ability to simulate brain-like cortical architecture and consequently, to contribute to our understanding of behaviorally relevant visual encoding. Incorporating topographic variations in feature encodings may allow neural networks to access priors contained in natural “embodied” visual experience (Mineault et al., 2021), built over million years of species-specific evolution. This would not only bring *in silico* models closer in line with *in vivo* visual systems, but it may also bridge the gap between species-specific visual inputs and artificial training sets, which lack the structure of natural embodied visual experience (Straub and Rothkopf, 2021). Alternatively, the use of more naturalistic “animal-view” movies as input (Betsch et al., 2004), coupled with architectural flexibility, may allow neural networks to learn topographic specializations predictive of patterns in the retina or other hierarchical layers of the visual system (Doshi and Konkle, 2021; Blauch et al., 2022). Recent work has shown some progress in this direction, with neural networks explicitly incorporating distinct objectives or constraints that result in the emergence of topographic organization and specializations (Plaut and Behrmann, 2011; Wang and Cottrell, 2017; Lee et al., 2020; Doshi and Konkle, 2021; Zhuang et al., 2021; Blauch et al., 2022; Konkle and Alvarez, 2022), shedding light on the origins of these organizational schemes.

## Conclusion

In summary, we have presented evidence for location-dependent feature sensitivity as an organizing principle of the entire visual system. This joint encoding framework contrasts with prevailing views that distinguish between representations of the location vs. features of objects in the visual field. Several studies (Milner and Goodale, 2008; McIntosh and Schenk, 2009; Cloutman, 2013; Milner, 2017) have shown that these pathways interact and the information streams are not segregated in the brain. Nonetheless, the two types of information are thought to be encoded by distinct circuits before they merge downstream. In contrast, our framework suggests that object location and identity are often encoded together by the same neuronal circuits, reflecting their inseparable existence in the visual field. We suggest that this principle reflects the evolutionary selection of sensory and motor circuits to optimally represent behaviorally relevant information suited to an animal’s unique sensory-motor demands. Future studies are necessary to discover mechanisms underlying joint encoding of location and functional information, how this relates to behavior, emerges during development, and varies across species.

## Author Contributions

MSS and DF conceived of the ideas in the manuscript, wrote the manuscript, and approved the submitted version.

## Conflict of Interest

The authors declare that the research was conducted in the absence of any commercial or financial relationships that could be construed as a potential conflict of interest.

## Publisher’s Note

All claims expressed in this article are solely those of the authors and do not necessarily represent those of their affiliated organizations, or those of the publisher, the editors and the reviewers. Any product that may be evaluated in this article, or claim that may be made by its manufacturer, is not guaranteed or endorsed by the publisher.
